# A Collaborative Brain-Computer Interface for Improving Human Performance

**DOI:** 10.1371/journal.pone.0020422

**Published:** 2011-05-31

**Authors:** Yijun Wang, Tzyy-Ping Jung

**Affiliations:** Swartz Center for Computational Neuroscience, Institute for Neural Computation, University of California San Diego, San Diego, California, United States of America; Cuban Neuroscience Center, Cuba

## Abstract

Electroencephalogram (EEG) based brain-computer interfaces (BCI) have been studied since the 1970s. Currently, the main focus of BCI research lies on the clinical use, which aims to provide a new communication channel to patients with motor disabilities to improve their quality of life. However, the BCI technology can also be used to improve human performance for normal healthy users. Although this application has been proposed for a long time, little progress has been made in real-world practices due to technical limits of EEG. To overcome the bottleneck of low single-user BCI performance, this study proposes a collaborative paradigm to improve overall BCI performance by integrating information from multiple users. To test the feasibility of a collaborative BCI, this study quantitatively compares the classification accuracies of collaborative and single-user BCI applied to the EEG data collected from 20 subjects in a movement-planning experiment. This study also explores three different methods for fusing and analyzing EEG data from multiple subjects: (1) Event-related potentials (ERP) averaging, (2) Feature concatenating, and (3) Voting. In a demonstration system using the Voting method, the classification accuracy of predicting movement directions (reaching *left* vs. reaching *right*) was enhanced substantially from 66% to 80%, 88%, 93%, and 95% as the numbers of subjects increased from 1 to 5, 10, 15, and 20, respectively. Furthermore, the decision of reaching direction could be made around 100–250 ms earlier than the subject's actual motor response by decoding the ERP activities arising mainly from the posterior parietal cortex (PPC), which are related to the processing of visuomotor transmission. Taken together, these results suggest that a collaborative BCI can effectively fuse brain activities of a group of people to improve the overall performance of natural human behavior.

## Introduction

Electroencephalogram (EEG) based brain-computer interfaces (BCI) in human studies have been demonstrated as a new tool to people with severe motor disabilities to communicate with their environments [1]–[3]. In recent years, the BCI community has devoted great effort to translating the BCI technology from laboratory demonstrations to daily-life applications [4]–[6].

Besides successes in the clinical research and practices in the past, researchers have been interested in applying the BCI technology to improving human performance [7], [8]. The concept of using BCIs for augmented cognition and action was first proposed in the early 1970s, and consequently led to different research topics. On one hand, some researchers sought to develop biofeedback techniques that would improve performance of military personnel engaged in tasks involving high mental loads [7]. On the other hand, some studies aimed to augment human-motor performance by sending control commands decoded from the brain without the delay for muscle activation [8]. Several recent studies have further demonstrated the feasibility of using BCIs to enhance human performance [9]–[20]. These studies can be categorized into four paradigms according to experiment designs and applications:

1. Motor action paradigm: Because a BCI establishes a direct link between the brain and the output device, the conventional pathway of peripheral nerves and muscles in motor control can be bypassed. Furthermore, delays between early stages of sensory information processing in other brain cortices (e.g., the visual cortex and the parietal cortex) and the stage of motor control in the motor cortex could be eliminated as well. Using this paradigm, motor behaviors can be predicted more rapidly than the actual motor reaction time (RT). For example, in the motor cortex, spatial patterns of movement-related cortical potentials (MRCPs) could be extracted to identify the body side of an upcoming movement (e.g., left hand or right hand) [9]. In our recent study, we showed that evoked EEG potentials in the posterior parietal cortex (PPC) could be used to predict directions of subsequent reaching or gazing movements [10].
2. Mental-state monitoring paradigm: Many studies have shown that signal changes related to alertness, arousal, and cognition are presented in EEG [11], [12]. Through capturing this kind of information in real time, the BCI technology can provide characterizations and understandings of human cognitive states, thereby improving human performance through sending warning feedbacks or controlling commands. Spectrum fluctuation is one of the EEG phenomena that have been widely used in EEG-based monitoring studies. In [13], changes of EEG power spectrum during a realistic driving task were used as a counter measure of alertness, which could be used to trigger auditory arousing feedback to effectively improve the driver's attention level. Error-related negativity (ERN) is another EEG signal, which has been successfully applied in BCI studies. In these systems, occurrences of ERN were considered as error indicators for correcting or cancelling incorrect manual/mental operations [14]–[15].
3. Visual target detection paradigm: Generally, target detection tasks need manual responses to confirm detections of targets. In practice, a target can also be indicated by brain activities such as a P300 event-related potential (ERP), which is elicited by a rare target event [16]. Therefore, only using a mental response arising from the brain can fulfill target detection. In an image based target detection task where mental response was employed, a rapid serial visual presentation (RSVP) paradigm could be used to improve human performance [17], [18]. For example, in [17], a real-time BCI system was developed to detect target images within enormous amount of images through detecting neural signatures of visual recognition events.
4. Additional input paradigms: Combined with traditional input devices, a BCI can provide an additional method to improve the speed of inputting. For example, when playing a video game, BCI-based controls combined with traditional input modalities (e.g., joystick, mouse, keyboard, etc.) could make operations faster [19]. Besides, in some specific conditions where conventional input methods are not accessible, a BCI can be used as an alternative approach. For example, in space applications, BCIs can provide hands-free interfaces for astronauts to facilitate operations in the condition with absence of gravity [20].

The concept of using BCIs to augment human performance is quite straightforward; however, this topic has not attracted much attention in real-life applications for ‘normal’ healthy populations due to technical limits of the EEG measurement and processing in real-world environments. One of the most challenging problems comes from the poor BCI performance caused by low signal-to-noise ratio (SNR) of EEG signals, making a BCI a frustrating alternative to other input modalities. For instance, the accuracies of a single-trial EEG classification using a binary finger-tapping task (left hand vs. right hand) in BCI Competition II ranged from 51% to 84% from 15 research groups [21]. Currently, the low accuracy of single-trial classification cannot satisfy performance requirements in many applications. To enhance the SNR, averaging methods have been widely used not only across multiple trials, but also across multiple subjects [22]. In ERP-based BCI systems, trial-based averaging is commonly used to improve system performance [23], [24]. In these systems, stimulus presentations were repeated many times, thereby producing multiple trials for the averaging process. However, in specific environments where real-time operations are necessary, the access to multiple trials from a single subject is not practical. Under these circumstances, fusing trials from multiple subjects can be used as an alternative. In human performance studies, it is a common sense that a team of individuals always outperforms individuals especially when performance requires multiple diverse skills, judgments, and experiences under time constraints [25]. Similarly, if single-trial EEG data from a group of people can be obtained and integrated, a better BCI performance could be expected. Based on this hypothesis, we propose a collaborative method for brain-computer interfacing, aiming to improve BCI performance through assessing collaborative brain activities from multiple users.

This study presents and discusses many issues in hardware/software designs and data processing for a collaborative BCI. First, it proposes a centralized paradigm and a distributed paradigm for system designs. Next, implementation of a collaborative BCI using a motor action paradigm is introduced. After that, three different data processing methods: (1) ERP averaging, (2) Feature concatenating, and (3) Voting, are proposed and compared in detail. Finally, this study demonstrates the efficacy of the collaborative BCI method in improving human performance.

## Methods

### 1 Ethics statement

The study was approved by the Human Research Protections Program of the University of California San Diego. All participants were asked to read and sign an informed consent form before participating in the study.

### 2 System diagrams for a collaborative BCI

**Figure 1:**
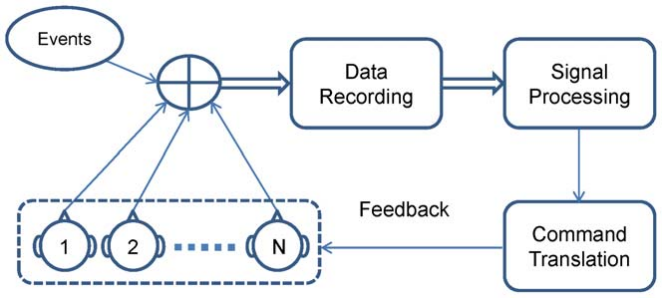
System paradigm of a collaborative BCI.

A collaborative BCI and a conventional BCI differ in many respects. A conventional BCI mainly aims to help the individual with motor disability to communication with the environment, whereas a collaborative BCI is specifically designed for improving human performance of healthy users. The basic design and operation of a collaborative BCI is shown in Figure 1. Similar to a conventional BCI [1], a collaborative BCI consists of three major parts: a data-recording module, a signal processing module, and a command translation module. Consequently, there are three major procedures in system operations. First, brain signals from a group of users are acquired by multiple EEG recording devices, and then are synchronized with common environmental events. Second, integrated EEG and event data are processed for extracting features for decoding users' intentions. Third, extracted features are directly translated to operation commands, which can also be used to provide sensory feedbacks to the users. Compared to a single-subject BCI, the complexity of system input from multiple users will lead to technical challenges in both data recording and signal processing procedures.

**Figure 2:**
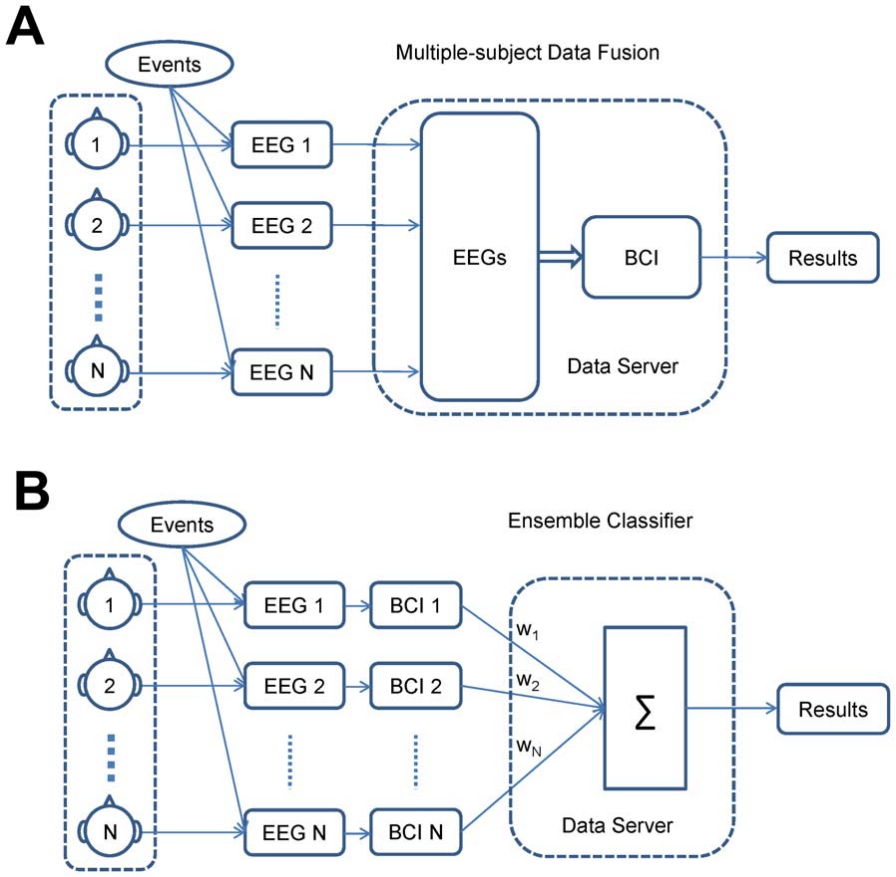
System diagrams for a collaborative BCI. (A) a centralized paradigm; (B) a distributed paradigm.

To implement a collaborative BCI, there are several specific requirements for hardware and software designs due to the employment of multiple users. First, multiple EEG recording systems need to work independently and simultaneously. Second, multiple-subject data need to be received and synchronized with respect to the common environmental events. Third, multiple-subject data recording and data processing procedures have to be performed in (near) real time. Ideally, the system can be implemented using a centralized paradigm similar to a conventional BCI (Figure 2a). In this paradigm, EEG data from multiple subjects are received and recorded, then thrown into a conventional BCI module for signal processing and command translation using a data server. A centralized paradigm is optimal for designing a collaborative BCI system; however, practicality of system implementation may be limited by heavy loads of data transmission and high computational costs caused by advanced signal processing and machine learning techniques [26], [27], [28], as well as low hardware/software robustness due to the involvement of multiple BCI subsystems.

To find a remedy to these problems associated with the centralized paradigm, we propose a distributed paradigm to facilitate the implementation of a collaborative BCI. As shown in Figure 2b, the whole system consists of multiple distributed BCI subsystems and a simplified data server. For each subject, a BCI subsystem works independently; each subsystem has its capability in EEG data acquisition and processing. In this paradigm, the amount of data transmitted between subsystems and the data server, as well as the computational cost for data processing, are significantly reduced. Because the data server only functions as an ensemble classifier for integrating classification results sent by the subsystems, the system robustness can be improved as well. The single-person BCI has been well studied in previous studies. Therefore this distributed paradigm is a more practical solution for implementing a collaborative BCI. The only disadvantage of the distributed paradigm is that costs of subsystem hardware might increase due to the employment of a data processing platform for each subject. In practice, portable data processing platforms such as a digital signal processor (DSP) platform can be integrated into the EEG recording device to reduce the overall system cost, and improve system practicality [29]. A collaborative BCI using the distributed regime can be considered a distributed computing system, in which each BCI subsystem solves the classification task independently in order to achieve a common goal (e.g., predicting motor response). Details of data analysis approaches for both paradigms will be discussed in the data analysis section.

### 3 BCI experiment

**Figure 3:**
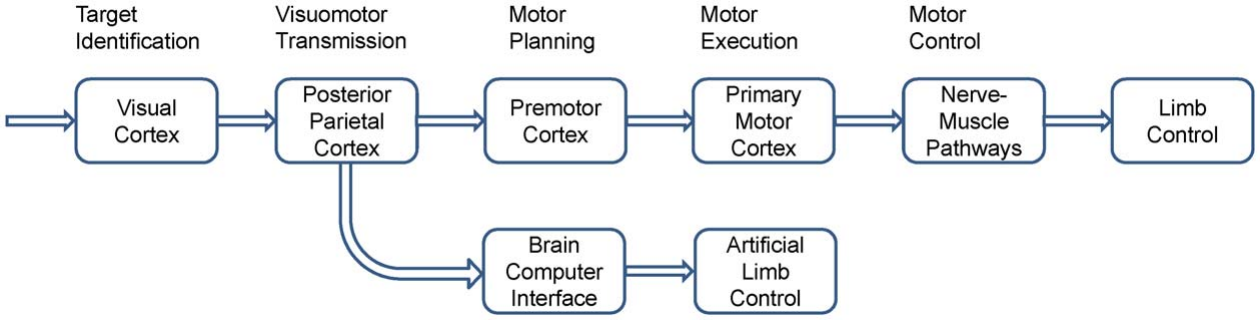
Information flow in a visuomotor control pathway and a BCI control pathway for a motor response.

This BCI study adopted a motor action paradigm reported in [10]. In the experiment, a visually guided reaching or gazing was employed as a motor response task. For the purpose of improving human performance, brain activities in the PPC, which occurred before actual motor response, were extracted for predicting the directions of upcoming movements. As shown in Figure 3, the response time (RT) of a cue-guided reaching movement consists of five stages: target identification, visual-motor transmission, motor planning, motor execution, and motor control. These processes occur sequentially in the visual cortex, the PPC, the premotor cortex, the primary motor cortex, and the nerve-muscle pathways. Through directly extracting embedded information from the PPC and bypassing the motor related procedures, this BCI system could accelerate a motor response by using an artificial limb.

#### 3.1 Subjects

An EEG and a behavior experiments were run separately on two groups of subjects. Twenty right-handed participants (12 males and 8 females, mean age 25 years) with normal or corrected-to-normal vision participated in the EEG experiment. Another group of 18 subjects participated in the behavior experiment (12 males and 6 females, mean age 23 years).

#### 3.2 Stimuli and procedure

A delayed saccade-or-reach task was used in the EEG study, allowing us to look for direction information in the EEG during the phase of movement planning. The experiment was comprised of nine conditions differing by movement types (saccade to target, reach without eye movement, or visually guided reach) and movement directions (left, center, or right). Each task was indicated to the subject by, first, giving an effector cue telling the type of action to be performed, followed by a direction cue and, finally, by an imperative action cue. Subjects were seated comfortably in an armchair at a distance of 40 cm from a 19-inch touch screen. A chin rest was used to help them maintain head position.

**Figure 4:**
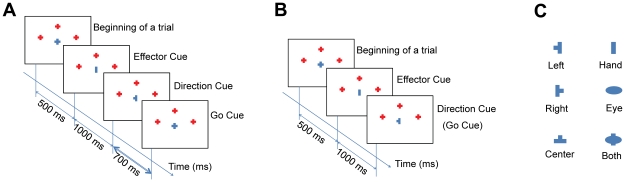
Time sequences of cue presentation and task-specific visual cues. (A) an EEG trial; (B) a behavior trial; (C) visual cues used to indicate effector and direction of a task.

Subjects used their right hands to perform the reaching tasks. At the beginning of each trial, the subject's forearm rested on the table with an index finger holding down a key on a keypad placed 30 cm in front of screen center. The sequence of visual cues in each trial is shown in Figure 4a. At the beginning of a trial, a fixation cross (0.65°×0.65°) was displayed at the center of the screen plus three red crosses (0.65°×0.65°) indicating potential target positions. The left and right targets had a vertical distance of 6° and a horizontal distance of 15° from the central fixation cross; the central target was 12° upwards. After 500 ms, an effector cue (0.5°×0.5°, rectangle, ellipse indicating hand and eye movements respectively, see Figure 4c) appeared at the screen center for 1000 ms. Next, a central direction cue (0.65°×0.65°, ⊣, ⊥, ⊢ for left, center, and right respectively) was presented for 700 ms. Subjects were asked to maintain fixation on the central cue until they started their responses, to perform the indicated response as quickly as possible following the disappearance of the direction cue (and reappearance of the fixation cross), and finally to return to their initial (key-down) position. There was a 400–600 ms interval for rest before the next trial started. Total trial duration amounted to 3500–4000 ms.

The behavior experiment was designed to measure the actual RT of a reaching movement using the same paradigm except that there was no delay after the direction cue, i.e., the direction cue was also used as the Go cue (see Figure 4b). In this experiment, subjects were instructed to response as fast as possible after the appearance of the direction cue.

#### 3.3 Data Recording

In the EEG experiments, EEG data were recorded using Ag/AgCl electrodes from 128 scalp positions distributed over the entire scalp using a BioSemi ActiveTwo EEG system (Biosemi, Inc.) referenced to the CMS-DRL ground. Eye movements were monitored by additional bipolar horizontal and vertical EOG electrodes. All signals were amplified and digitized at a sample rate of 256 Hz. Three cue presentation events and two manual response events (“button release” and “screen touch”) were recorded on an event channel synchronized to the EEG data by DataRiver software (A. Vankov). EEG and behavioral data were recorded from 20 subjects on different days using the exactly same target presentation sequences. Some practice blocks were run before starting the EEG recording. For each subject, the experiment consisted of four blocks (with breaks in between) each including five runs of 45 trials. Within each block, there was a 20-second rest between runs. A total of 900 trials were equally distributed between the nine tasks, which were presented to the subject in a pseudorandom sequence.

In the behavior experiment, only the events were recorded for obtaining the actual RT for a reaching response. For each subject, the experiment consisted of three blocks with a total of 675 trials equally distributed among the nine tasks.

#### 3.4 Data preprocessing

This study focused on the estimations of planned direction of movement. For simplicity, we only used “left” and “right” conditions for “hand” tasks for further analysis. The same analysis could be applied to data under “eye” and “both” conditions. Epochs from the response delay period, 0 to 700 ms following the onsets of direction cues, were extracted from the continuous data, and labeled by movement directions. The period [−100 ms 0 ms] was used as the baseline for each trial. Electrodes with poor skin contact were identified by their abnormal activity patterns and then removed from the data.

**Figure 5:**
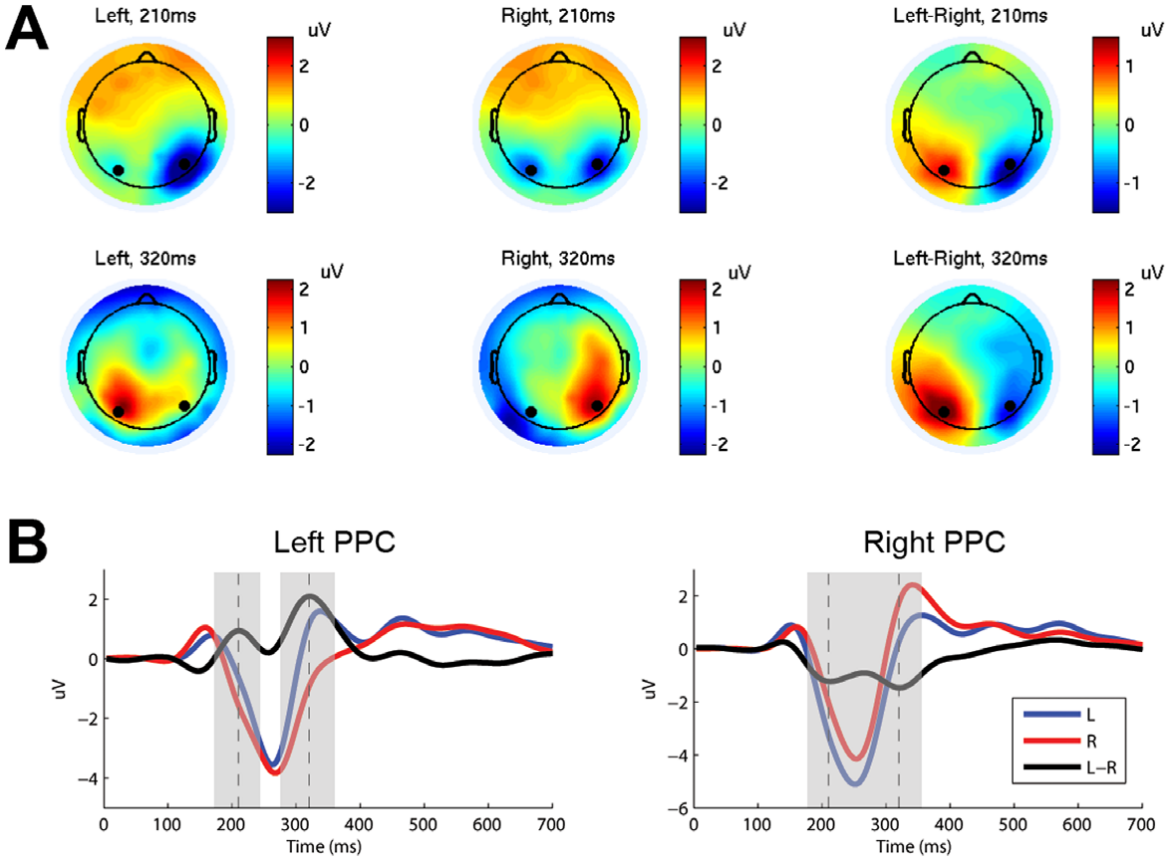
Scalp maps and temporal waveforms of ERP signals. (A) Grand average 128-channel scalp maps of ERPs and difference waves (*left*-*right*) across all subjects at 210 ms and 320 ms. Black dots indicate positions of two selected electrodes near the PPC. (B) Average ERP waveforms across all subjects on two PPC electrodes in *left*- and *right*-reaching conditions and their difference. Dash lines mark peaks of difference waves at 210 ms and 320 ms. ERPs at two PPC electrodes show significant differences between *left* and *right* conditions using a paired t-test across subjects (left PPC: p<10^−5^ at 210 ms and p<10^−6^ at 320 ms, right PPC: p<10^−6^ at 210 ms and p<10^−4^ at 320 ms). The shaded intervals indicate areas where differences between *left* and *right* conditions are significant (p<0.05).

We used independent component analysis (ICA) as an unsupervised spatial filtering technique to remove artifacts arising from eye and muscle movements. For each subject, all trials were band-pass filtered (1–30 Hz), concatenated, and then decomposed using the EEGLAB toolbox [30]. To retain the low-frequency EEG activities, ICA weights of the decomposition were applied to original unfiltered data before artifact removal. To extract the direction-specific activity of the ERPs, we compared the spatiotemporal patterns of EEG corresponding to different movement directions. As shown in Figure 5, we found a hemispheric asymmetry over the parietal cortex during the delay period (0–700 ms) in which motor planning can be presumed to have continued until the cued movement onset (appeared after 700 ms). Two lateral electrodes representing PPC activities showed a significant contralateral negativity and ipsilateral positivity with respect to the intended movement direction (Figure 5b). Across all subjects, difference waves between reaching left and reaching right conditions showed two peaks located at 210 ms and 320 ms after the direction cue. ERP scalp maps of two conditions and their difference at these two selected frames were illustrated in Figure 5a.

#### 3.5 Feature extraction and classification

**Figure 6:**
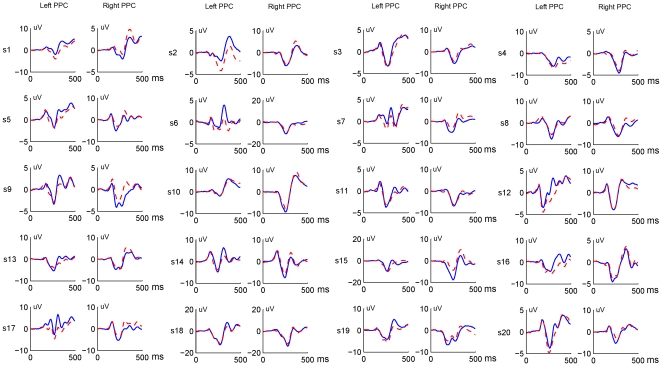
Grand average ERPs at electrodes placed at the left and right sides of the PPC for 20 subjects. Solid lines indicate the reaching *left* condition, and dash lines indicate the reaching *right* condition.

The goal of this study is to demonstrate the efficacy of a collaborative BCI, rather than the EEG dynamics associated with all different task conditions. Therefore, the analysis below focuses only on the classification performance of predicting the intended movements based on the directional EEG information generated in the parietal cortex. To this end, two lateral electrodes over the PPC areas were selected for feature extraction based on the significance of ERP difference between left and right conditions. Figure 6 shows ERP waveforms at two PPC electrodes for all subjects. The direction-related asymmetry in the PPC was highly reproducible across subjects. Through time-frequency analysis, we found that the ERP difference was mostly contributed by EEG components with a frequency band lower than 12 Hz. To reduce feature dimension, EEG signals were downsampled by calculating the mean of five continuous data points without overlapping. For feature extraction, within a selected time window, EEG amplitudes were normalized at each time point to have a range of [−1 1] across trials and conditions, and then normalized amplitudes from two PPC electrodes were concatenated into a feature vector:
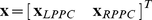
(1)


For classification, a support vector machine (SVM) classifier with an RBF kernel was implemented in the MATLAB® Bioinformatics Toolbox. The RBF kernel was optimized according to average classification performance across all subjects. To facilitate the training procedure, the scaling factor in the RBF kernel was fixed at 10 for all SVM classifiers. In this study, 10×10-fold cross validation was run to estimate classification performance for all classification tasks.

### 4 Collaborative BCI data analysis

For each subject, classification of “left” versus “right” trials was performed using a standard machine-learning paradigm. For a collaborative classification based on data from multiple subjects, we propose three approaches to fuse the information from multiple subjects: (1) ERP averaging across subjects, (2) feature combination (e.g., concatenating features from multiple subjects), and (3) voting using an ensemble classifier. All these approaches can be implemented in the centralized paradigm, but for the distributed paradigm, only the voting approach is practical because data from each subject are processed separately in each of the BCI subsystems.

#### 4.1 ERP averaging

A widely used method for analyzing ERP has been to average EEG measurements over an ensemble of trials within a subject or across subjects [22]. Ensemble averaging can enhance the SNR of ERP given a linear mixing model: 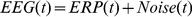
(2)


where *ERP*(*t*) is a constant signal (i.e., the evoked brain response) and *Noise*(*t*) is a random signal with zero mean (i.e., the background EEG activity) in different trials. In a collaborative BCI system, multiple trials can be obtained through collecting single-trial data from multiple subjects. Therefore, the ensemble averaging method can be implemented across subjects:
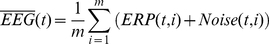
(3)


where *i* is subject index and *m* is the total number of subjects.

#### 4.2 Feature combination

According to ERP studies, the model in equation (2) is not true when considering a more complicated ERP model, which involves multiple components [22]: 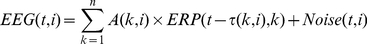
(4)


where *ERP* is assumed to consist of *n* components, with independent amplitude modulation indicated by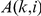 and latency jitter indicated by 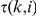. Under this circumstance, ensemble averaging might lose information due to individual differences among subjects. For example, latency jitter might cancel out ERP signals when two adjacent components have different polarities. Therefore, to maintain intact information from all the subjects, the feature combination method might be more suitable for a collaborative system.

In the machine learning theory, feature combination can improve overall classification accuracy due to independence between features. Recently, following the wide employment of machine learning techniques in BCI studies, feature combination methods have been introduced in EEG classification [5], [27]. For simplicity, we use a feature concatenating method, which is easy to implement:
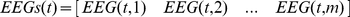
(5)


where the combined feature vector is a concatenation of feature vectors from *m* subjects.

Theoretically, feature combination is optimal for a collaborative BCI. However, considering the fact of a BCI system that training data is always limited and feature combination will significantly increase the dimensionality of feature space, the feature combination method might encounter an overfitting problem. For example, the dimension of features from a single subject is 50 in equation (1) when using the time window of [0 ms 500 ms], which will be increased to 1000 for 20 subjects. However, the number of the training samples remains the same as in the single subject condition (100 trials per condition). Therefore, the performance gain of feature combination will be weakened due to a small training-set size.

#### 4.3 Voting

Ensemble classifiers have been widely used in the area of machine learning [31]. An ensemble classifier consists of multiple sub-classifiers and a voting system. In the case of a binary classification where two classes are labeled as +1 and −1 respectively, the procedure for a weighted voting can be described as follows:
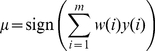
(6)


where *w, i.* is the subject specific weight and *y, i.* is the output of a sub-classifier. In our study, an SVM classifier was trained as a sub-classifier for each subject, and the training accuracy was used as the voting weight.

As mentioned before, the voting method is the only solution for a collaborative BCI using the distributed paradigm. Ideally, if there is no interaction between subjects, the voting method is supposed not to lose useful information for classification.

#### 4.4 Training and testing

Realization of training and testing procedures of a collaborative BCI depends on the method used in feature extraction. The ERP averaging and Feature concatenating methods have to be realized on a centralized computer infrastructure where original EEG data from different subjects can be collected and processed. The Voting method can be realized either on a centralized or a distributed system.

In the collaborative BCI regime, a ‘single-trial’ actually comprised multiple epochs from multiple subjects following the same task stimulus. A 10×10-fold cross validation was used to assess classification performance. For the ERP averaging method, features of each trial were obtained by averaging feature vectors (Equation (1)) across subjects. An SVM classifier was then trained with the training set and applied to classification of the testing set. The Feature concatenating method used a similar way except that features were obtained by concatenating feature vectors from individuals (Equation (5)). In the Voting method, an SVM sub-classifier was used for training and testing for each subject separately. The collaborative classification was then performed using Equation (6).

#### 4.5 Number of subjects

The number of subjects is an important parameter for a collaborative BCI. In general, more subjects can provide more information for improving classification. Generally, when average performance is poor, any subject who has classification accuracy higher than the chance level can improve the overall performance of a collaborative BCI. However, the system costs (including hardware, software, and human resources) will also increase when more subjects are involved. Therefore, a tradeoff between the system performance and system cost should be made according to the specificity of the application.

To answer the question of how many subjects are needed to implement a satisfactory collaborative BCI, we evaluated system performance with respect to the number of subjects. For each number *n* (from 1 to 20), a random combination (*n* out of 20 subjects) was repeated 500 times for calculating classification accuracy using cross validation. All of the three collaborative approaches were calculated for comparison using data within the time window of [0 ms 400 ms]. A one-way Analysis of Variance (ANOVA) was used to investigate the effect of ‘number of subjects' on classification performance. Furthermore, for each collaborative condition (*n* from 2 to 20), the two-sample T-test (500 samples for the collaborative method vs. 20 samples for the individual method) was used as a post-hoc test to evaluate if the performance of a collaborative BCI was significantly better than that of an individual BCI.

#### 4.6 Prediction time

In an application regime such as a target detection task, response time is always a critical parameter for evaluating human behavioral performance. In the motor action paradigm used in this study, we aimed to improve human performance through accelerating a motor decision-making, compared to RT. Therefore, it would be interesting to find out how fast a collaborative BCI can predict the direction of an upcoming reaching movement.

The actual mean RT for the hand reaching tasks measured in the behavior experiment was 464±62 ms across 18 subjects. As discussed before, response direction can be determined through extracting brain activities related to the visuomotor transmission procedure. According to prediction time, the system improves the overall performance when response direction can be accurately predicted at any time point earlier than the RT. To explore the system's capability of accelerating motor decision-making, we evaluated the system performance at different time durations used for feature extraction. Time windows with zero onset and different offsets starting from 100 ms and ending at 500 ms, incrementing with an interval of 10 ms, (i.e., 0–100 ms, 0–110 ms, …, 0–500 ms) were used to calculate accuracy-time curves. To show interaction between the prediction time and the number of subjects, different numbers of subjects (1, 5, 10, 15, and 20) were included for comparison.

## Results

**Figure 7:**
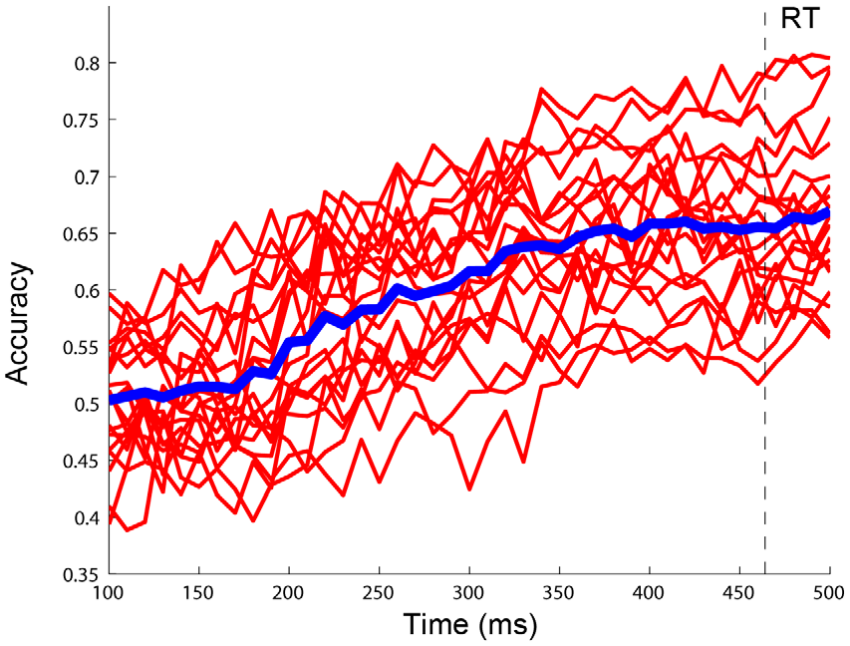
Time course of single-subject classification accuracy. Thin solid lines indicate classification results for 20 subjects, and the thick solid line shows the averaged accuracy. The dash line indicates the mean response time (RT) measured in the behavior experiment (464 ms).

Figure 7 shows the accuracy of single-trial classification for all 20 subjects using a single-subject classification paradigm. The classification accuracy increased in accordance to the increase of the time window length used for feature extraction. With window length shorter than 150 ms, the average accuracy was around the chance level (mean±standard deviation: 51.1±5.9%). After 150 ms, the accuracy increased gradually and reached 67.0±7.5% at 500 ms. The tendency of performance improvement is consistent with the differences in time courses of ERP waves between left and right conditions, which reflect temporal dynamics of the PPC activities during directional movement planning. There was a large individual variability in single-trial classification accuracy: <60% in four subjects, 60–70% in 10 subjects, and >70% in six subjects. These results indicate that EEG activities near the PPC can provide useful information for predicting the intended movement direction. Although single-trial classification performance for single subject is low, it provides a substantial basis for building a collaborative BCI.

**Figure 8:**
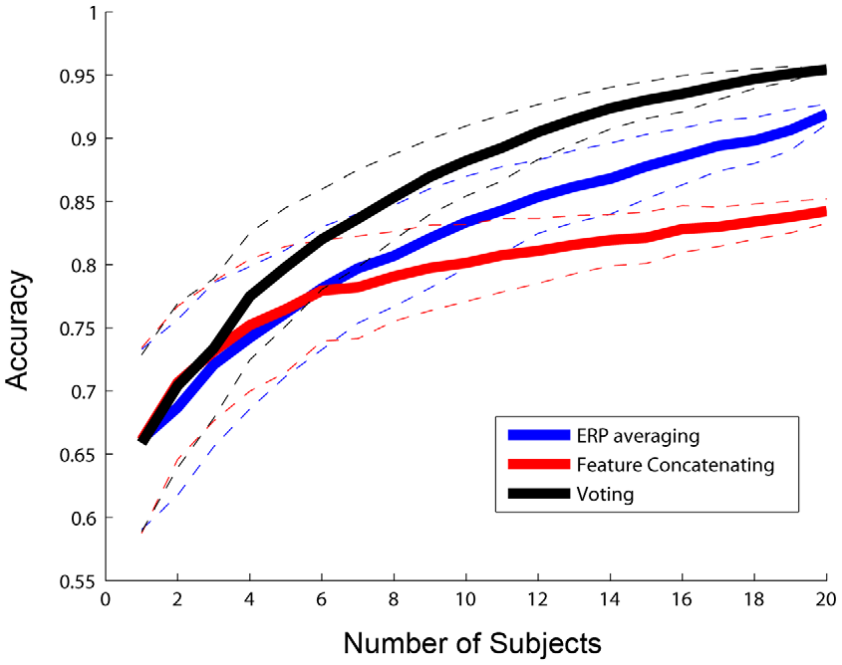
Classification accuracy of different collaborative classification methods as a function of the number of subjects. Solid lines indicate mean accuracy, and dashed lines indicate mean accuracy ± standard deviation.

Theoretically, the collaborative classification is expected to achieve a significant gain in the overall system performance. Figure 8 illustrates the prediction accuracy for the three collaborative methods as a function of the number of subjects. Three major findings are summarized as follows:

1. Classification performance for all three collaborative methods had been significantly improved when data from multiple subjects were combined and integrated. The ANOVA showed a highly significant effect of ‘number of subjects' on classification performance (Voting: F(19, 9980) = 3061.83, p = 0.00; ERP averaging: F(19, 9980) = 1634.06, p = 0.00; Feature concatenating: F(19, 9980) = 809.35, p = 0.00). When data of two subjects were combined, the T-test showed a significant difference between the individual performance and the collaborative performance when using the Voting method (p<0.01) and the Feature concatenating method (p<0.001) respectively. For the ERP averaging method, at least three subjects were required to reach a significant level (two subjects: p>0.05, three subjects: p<0.0001). A more prominent significance was obtained when the number of subjects increased. Although classification accuracy for single subject was low (mean across subjects: 66%), the collaborative method could still reach a high classification performance. For example, when using all 20 subjects, all three methods showed significantly improved accuracy (95% for the Voting method, 92% for the Averaging method, and 84% for the Feature Concatenating method).
2. The classification accuracy was enhanced substantially as well as the standard deviation decreased when the number of subjects increased. For example, using the Voting approach, the accuracy increased from 66% to 80%, 88%, 93%, and 95% as the number of subjects increased from 1 to 5, 10, 15, and 20, respectively, meanwhile, the standard deviation reduced from 7.0% to 1.0% when the number of subjects increased from 1 to 20. These results proved the existence of independence between subjects, which made all subjects contribute to the improvement of system performance and robustness.
3. The Voting method is optimal for collaborative EEG classification. The Voting method always outperformed the ERP averaging method when multiple subjects were involved. Accuracy of the Feature concatenating method was obviously affected by the overfitting problem. As shown in Figure 8, when the number of subjects was small (<4), the Feature concatenating method and the Voting method showed similar results, both outperformed the ERP averaging method. When the number of subject increased above 4, the Voting method outperformed the Feature concatenating method. Further, when the number of subjects was above 6, the Feature concatenating method was even worse than the ERP averaging method. When using all 20 subjects, the ERP averaging method had accuracy much higher than the Feature concatenating method (92% vs. 84%).

**Figure 9:**
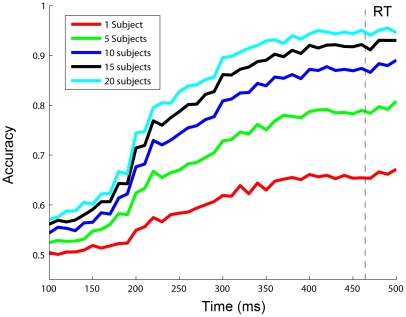
Classification accuracy of different numbers of subjects as a fuction of the window length. The dash line indicates the mean response time (RT) measured in the behavior experiment (464 ms).

As mentioned before in the method section, time required to make a prediction is a very important parameter to evaluate the performance of a BCI system in a motor action paradigm. Figure 9 shows the classification accuracy as a function of the length of time windows used for data analysis. Results for 1, 5, 10, 15, and 20 subjects were put together to show the interaction between the number of subjects and the prediction time. The results clearly showed that the acceleration of decision-making depended on both the desired accuracy and the number of subjects involved in the collaborative system. For example, when an accuracy of 70% was required, decisions could be made at 200 ms by 20 subjects, which was around 250 ms ahead of subjects' actual responses. If 95% was required, the prediction time had to be extended to 400 ms. Concerning the number of subjects, the decision could be made faster with more subjects when the same classification accuracy was required. For example, toward an accuracy of 70%, 280 ms and 200 ms were required for 5 and 20 subjects, respectively.

## Discussion

### 1 System performance

This study demonstrated that a collaborative BCI could significantly improve system performance through integrating useful information from a group of users. Obviously, system performance can be further improved if more subjects are involved in the system, however the system cost and complexity also increase accordingly.

This study also explored three methods for fusing and analyzing collaborative EEG from multiple subjects. The results of this study showed that the Voting method was optimal for collaborative EEG classification, while all three collaborative BCI outperformed the single-subject BCI.

### 2 Online system implementation

Currently, there are several challenges that have to be resolved before an online collaborative BCI system can become a reality. First, a collaborative BCI needs multiple BCI hardware platforms, which consist of an EEG recording system and a real-time signal-processing platform. Because commercial EEG products used for EEG research are still expensive, the total cost for building a collaborative BCI will be high. In practice, low-cost and customized EEG recording devices and signal-processing platforms can be used for implementing a collaborative BCI. Second, a collaborative system requires specific software development. As mentioned in the method section, the system needs seamless communication between EEG systems and signal-processing platforms, and between the BCI subsystems and the data server. Furthermore, data processing in BCI subsystems and the data server has to be implemented in (near) real time. Third, the complexity of a collaborative BCI needs to be further reduced to reality. When using conventional wet electrodes, capping and user training will be very labor intensive and time consuming. In a collaborative BCI regime, these procedures need to be simplified considerably.

Although the system demonstrated in the current study was implemented in an offline manner, it can be directly transferred to an online system if the hardware and software requirements can be met. With advances in biomedical electronics and telecommunication technology, it will soon be possible to implement an online collaborative BCI system. Recently, Wang et al. [32] have designed and implemented a mobile wireless BCI platform using a wearable EEG recording device and a Bluetooth enabled cell-phone. This platform can provide a practical solution for implementing a collaborative BCI system. A pilot study of a collaborative BCI system using the cell-phone based BCI platform is currently undertaken. Furthermore, several recent studies have demonstrated the possibility of using dry electrodes and zero-training methods to facilitate the use of a BCI system [33], [34]. These new technologies can also be employed into an online collaborative BCI system.

### 3 Potential applications

This study demonstrated an application of the collaborative BCI to accelerate motor decision-making of a reaching movement. Moreover, a collaborative BCI can be applied to many other applications in which the overall human-system performance is critical. It will be especially useful for real-time situations where classification accuracy is critical, but performance of single-user BCI is poor.

A collaborative BCI system can also be used as a platform for studying the human brain in naturalistic environments. For example, using a collaborative BCI system, social interaction involving a group of people can be studied with real-time monitoring of brain activities to explore the underlying brain mechanisms.

In addition, other emerging applications of BCI's such as classroom education, neuroeconomics, and video gaming might also benefit from a collaborative BCI. A collaborative BCI might enhance the effectiveness of training and educational programs through monitoring either the student's attention/concentration or ability to participate effectively. Similarly, it can be applied to the field of neuroeconomics to evaluate the effects of designs of advertisements on the brain activities. Recently, the BCI technology has also been introduced into video gaming [19]. It is foreseeable that the employment of a collaborative BCI will make multiple-role games more attractive for players.

### 4. Future directions

Future directions to improve single-subject performance include:

1. Using more electrodes: Despite that all 128 channels were used in the procedure of ICA-based artifact removal, two electrodes placed near the PPC areas were selected for feature extraction. The system performance might be improved if more electrodes are employed. Firstly, for more electrodes over the PPC area, spatial filtering techniques can be applied to improve the SNR through removing task irrelevant activities [35], [36]. Secondly, electrodes located over other brain areas might provide additional information for decoding movement intention. For example, movement intention could also be decoded based on EEG activity in the premotor and the motor cortices, which are known to play important roles in movement planning and execution.
2. Using subject-specific parameters: For simplicity, this study used the same time-frequency parameters for different subjects, which might not be optimal due to individual variability. It has been shown in previous studies that subject-specific parameters can significantly improve classification accuracy of a BCI [37]. Through optimizing parameters in the time, frequency, and space domains, classification performance for each subject might be improved.
3. Adding additional EEG features: This study only used ERP amplitudes as the features for classification. Other features such as spectral modulation might be a complementary feature for improving the classification performance. For example, Thut et al. [38] showed that the direction of visuo-spatial attention could be predicted by measuring alpha-band power over the two posterior hemispheres.

Future directions for improving the overall collaborative BCI system include:

1. To improve the ERP averaging method, weighted averaging methods might be helpful for enhancing the SNR of EEG signals [39].
2. Improvement of the Feature concatenating method might be achieved from several directions, aiming to reduce overfitting. First, the dimension of the features can be reduced using feature selection methods. Second, generalization ability can be improved through increasing the size of training data. Third, classifiers specifically designed for high-dimensional data with a small training set might be helpful.
3. To improve the Voting method, the effort should be put on using other ensemble classifiers with better performance. Besides, some other ensemble learning techniques, such as the boosting and the bagging methods [31], might be useful to improve the robustness of the collaborative classification.

### Conclusion

This study proposed a collaborative BCI paradigm, which fused single-trial EEGs from multiple subjects to improve the overall BCI system performance. By comparing system designs and data analysis methods, this study showed that a distributed paradigm combined with a Voting classifier is a practical solution for implementing a collaborative BCI system. The feasibility and efficacy of the proposed BCI system was demonstrated through a collaborative BCI that could accelerate motor decision-making of a cue-guided reaching movement. The classification accuracy of the system showed a significant improvement over that of the single-subject BCI. Furthermore, the collaborative BCI allowed the subject's reaching direction to be estimated much earlier than his/her actual motor response. In summary, this study designed and demonstrated the use of the collaborative BCI technology to improve human performance in natural environments.
